# PHYLOViZ Online: web-based tool for visualization, phylogenetic inference, analysis and sharing of minimum spanning trees

**DOI:** 10.1093/nar/gkw359

**Published:** 2016-04-29

**Authors:** Bruno Ribeiro-Gonçalves, Alexandre P. Francisco, Cátia Vaz, Mário Ramirez, João André Carriço

**Affiliations:** 1 Instituto de Microbiologia and Instituto de Medicina Molecular, Faculdade de Medicina, Universidade de Lisboa, 1649-028 Lisboa, Portugal; 2 INESC-ID / Instituto Superior Técnico, Universidade de Lisboa, 1000-029 Lisboa, Portugal; 3 Instituto Politécnico de Lisboa, 1959-007 Lisboa, Portugal

## Abstract

High-throughput sequencing methods generated allele and single nucleotide polymorphism information for thousands of bacterial strains that are publicly available in online repositories and created the possibility of generating similar information for hundreds to thousands of strains more in a single study. Minimum spanning tree analysis of allelic data offers a scalable and reproducible methodological alternative to traditional phylogenetic inference approaches, useful in epidemiological investigations and population studies of bacterial pathogens. PHYLOViZ Online was developed to allow users to do these analyses without software installation and to enable easy accessing and sharing of data and analyses results from any Internet enabled computer. PHYLOViZ Online also offers a RESTful API for programmatic access to data and algorithms, allowing it to be seamlessly integrated into any third party web service or software. PHYLOViZ Online is freely available at https://online.phyloviz.net.

## INTRODUCTION

High-throughput sequencing methods gave rise to a paradigm shift in microbial typing and genomic population structure studies (1,2). The ability to partially sequence the genomes of hundreds to thousands of strains created the need for effective ways to represent relationships between strains that are scalable and robust. Single Nucleotide Polymorphism (SNPs) analysis and whole or core genome MultiLocus Sequence Typing (wgMLST or cgMLST) (3), result in profiles that have thousands of loci which can be used for outbreak investigation, epidemiological surveillance of clones of interest and bacterial population or evolutionary studies. These profiles can be analyzed using traditional phylogenetic algorithms or minimum spanning tree (MST) like approaches (4,5). The latter are particularly suited to cope with the increasing number of strains used in each study, since most phylogenetic analysis methods can be time consuming for large numbers of strains or require high performance computing facilities not available to most users.

PHYLOViZ software (6) was developed as a platform to incorporate phylogenetic data analysis from multiple data sources with the possibility of annotating the resulting tree with epidemiological data. PHYLOViZ was designed with the understanding that data visualization and integration of multiple data sources was crucial to obtain insights and formulate new hypothesis, particularly regarding epidemiology and outbreak investigation of microbial pathogens. The interactive displays of information, where the user can quickly switch between the combinations of parameters being represented, allows for the kind of analytical reasoning proposed by the visual analytics agenda (7). However, PHYLOViZ lacks options to exchange visual representations between users or to provide access to a given dataset for exploration by other users. PHYLOViZ was created using cross-platform JAVA, but runs on the user computer while data sharing is facilitated by web applications that do not require the recipient to have any particular software installed. A few tree visualization and annotation tools allowing data sharing and integration of epidemiological data are available (8–11). However, these only use information from pre-defined trees and most are not focused in developing approaches to improve comparative analyses.

With the aim of overcoming these limitations, PHYLOViZ Online was developed as a user-friendly web application for profile-based data analysis, visualization and sharing, also allowing the application of visual analytics processes on trees defined previously through traditional phylogenetic methods.

## ALGORITHMS AND SOFTWARE

### Input data types

PHYLOViZ Online accepts three types of data as input. (i) Profile data in a Tab-delimited file format, containing profile data from sequence based typing methods such as traditional Multilocus sequence typing (MLST), cgMLST, wgMLST (including gene presence or absence), Multilocus variable-number tandem repeat analysis (MLVA) or SNPs. Descriptive headers in the first row are required and the first column must have profile identifiers for each strain. Each of the subsequent rows represents the information for an individual strain. (ii) FASTA files with sequences of the same length or aligned to the same length. Each character is compared to the same position on other sequences and distances are computed using Hamming distance, i.e the number of differences between sequences. This file format can be used to analyze SNP data. (iii) Newick format files with tree topology and branch lengths. In this file format, each branch has to have an identifier in order for it to be represented by PHYLOViZ Online. Absent branch lengths will be represented as branches of a minimal pre-defined length.

Users can also provide a file with auxiliary data in tab-delimited format to be represented onto the tree, such as demographic, temporal or epidemiological information, including antibiotic resistance or typing information from other methods. The link between the data and the auxiliary data depends on the initial input file type. Identical column headers in the profile and auxiliary data files will identify the location of the information used to link the sources, while for FASTA and Newick data, identifiers from the two files types will be searched in the first column of the auxiliary data to link the sources.

### Implementation

PHYLOViZ Online is a Node.js (https://nodejs.org/) application developed in a modular perspective, separating data storage and management from data processing and visualization. Information provided by users from different input formats is processed into JavaScript Object Notation (JSON) and stored in a PostgreSQL relational database (version 9.4). Communication between server and client is made using a developed RESTful API, which allows programmatically to perform queries to the database and manage data, which will then be used in visual analytics. The application is freely available to any user through a web-browser and there is no login requirement. The application is fully supported in Google Chrome Version 49.0.2623.110. Safari Version 9.0.3 and Firefox Version 45.0.1, currently support all features, but with some performance loss. Without a login, users have full access to PHYLOViZ Online capabilities but any shared data will only persist for 24 h. Authenticated users, have access to a private area to upload and store their own data, with the option of sharing data through permanent links and to make a dataset publicly available.

The web application is divided into five tabs with four types of visualizations: Tree visualization (Tree tab), Tables (Primary and Auxiliary data tabs), Interactive Distance Matrix (Distances Tab) and Sequence Viewer (Sequences Tab).

When a profile or FASTA input is used, the goeBURST algorithm (5) generates an MST like representation, using a set of tie-break rules based on the number of locus differences for each strain. The resulting tree visualization is done using VivaGraphJS’ (https://github.com/anvaka/VivaGraphJS) force directed layout. Visualization rendering is assigned to the WebGL JavaScript API, which allows visualization of thousands of nodes in the web browser by taking advantage of GPU hardware acceleration widespread in laptops and desktops. Tables are generated using the DataTables (https://www.datatables.net/) JavaScript library, allowing for querying, selection and data export. A visual representation of multi-sequence alignment for FASTA input files was also implemented using the BioJS MSA Viewer package (https://www.npmjs.com/package/msa), which allows to explore sequences, order and highlight regions according to data characteristics. Additional visual representations (Pie Charts and Interactive Distance Matrix) are constructed using Data Driven Documents (D3.js) (https://d3js.org/).

There is communication between the four distinct visualization parts. Fields from tables can be selected and represented graphically onto the tree. When a table field is selected, an additional global Pie Chart that associates counts and colors to the selected values is created. These colors are then used when representing the table information onto the tree.

The Interactive Distance Matrix and the Sequence Viewer are not available by default but they can be created and redone at any time. Users are able to select sets of nodes from the Tree visualization in order to calculate custom interactive distance matrices and visualize multi-sequence alignments, when applicable. These allow a graphical representation and exploration of the actual pairwise distance between the selected strains.

### Data visualization

#### Tree visualization

**Figure 1.**
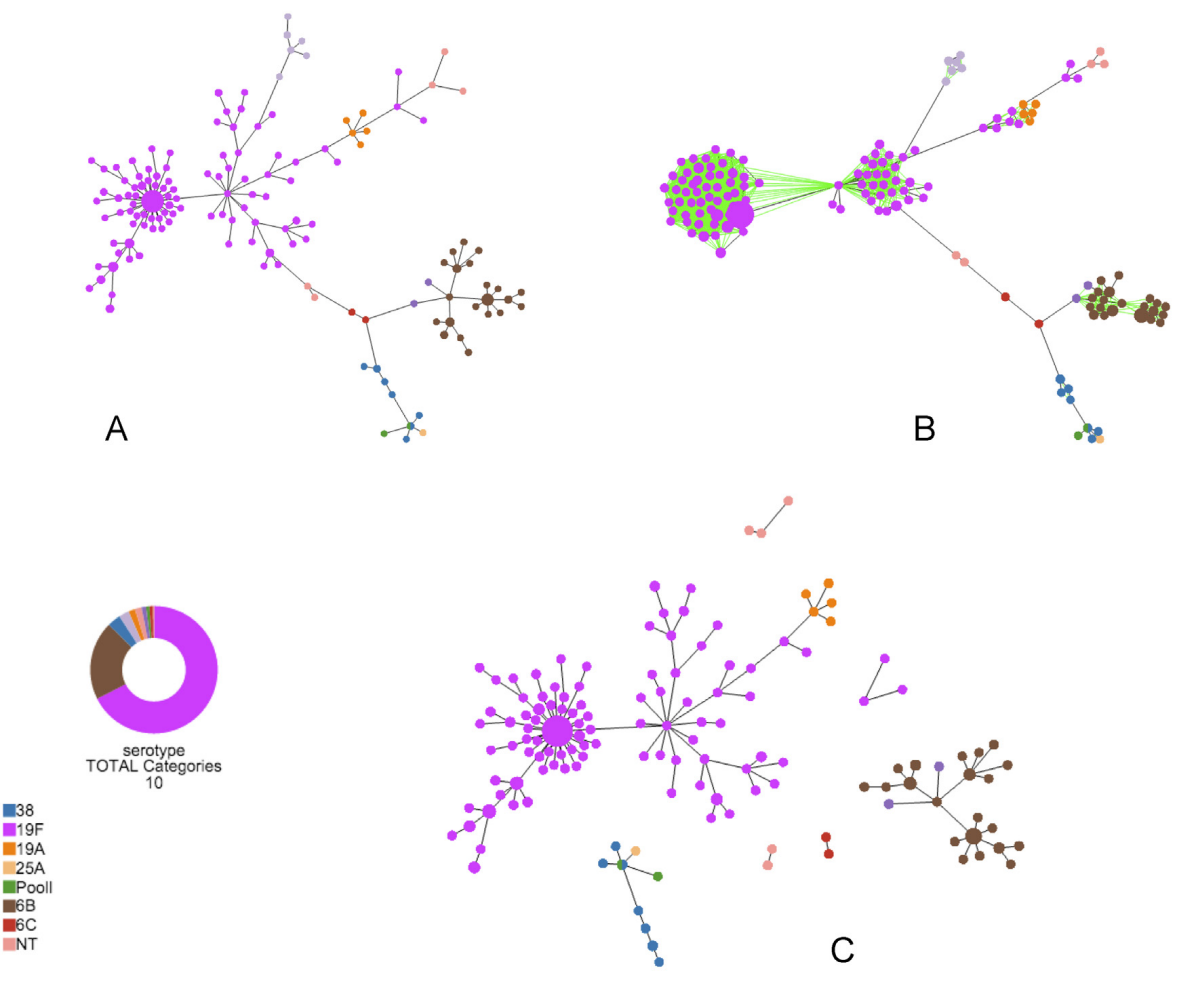
Different features available at *Tree Visualization*. (**A**) MST of a *Streptococcus pneumoniae* cgMLST dataset. Colors were attributed to nodes according to the serotypes existing in the auxiliary data file. (**B**) N Locus Variant graph. All nodes with distances equal or above 12 were linked. It is possible to visualize two distinct clusters in the 19F serotypes at a level of 12 differences between profiles. At that level, 6A and 6B serotypes are also clustered together. (**C**) Tree cut-off algorithm. All links from the MST with a distance value above 60 were deleted. Clusters are defined with different serotypes.

Tree visualization is the default starting tab in the analysis. Colors can be assigned according to loci in allelic profiles (Profile files), sequence position (FASTA files) or auxiliary data (Figure 1A). Each node will become a colored pie chart, reflecting the distribution of strains with different values for the fields selected represented by each node. Users can manually select the colors by clicking in the color legend.

In order to better explore the resulting tree and the relationships between strains, two different operations that modify the default characteristics of the MST are available to the user: the N Locus Variant (NLV) (Figure 1B) graph and a Tree cut-off threshold (Figure 1C). The NLV graph easily identifies sets of closely related nodes by relaxing the MST construction restriction, allowing the display of all possible links up to a specific threshold (ranging from 0 to the maximum number of differences between nodes). The Tree cut-off threshold splits the MST by removing links above a certain value ranging from 0 to the maximum number of differences. In any of the two operations, the user can restore the original MST by returning threshold values to the default state. These features are also available for Newick trees defined as Cluster Nodes and Tree cut-off operations, which allows to link all nodes with a branch length below the value specified by the user or remove the links above a certain branch length.

Users are also given the ability to save the tree layout, which is particularly useful and time-saving when working with large trees.

#### Tables

In addition to the Tree Visualization, PHYLOViZ Online also displays input data in a tabular format. It shows Primary data for profile-based input and any Auxiliary data. Users can filter information by performing queries on the data table. Queries can use regular expressions (regex).

Tables interact with the Tree visualization. Upon column selection, a Pie Chart is constructed based on the selection. The ‘Link to Tree’ button allows users to directly transfer the selection of specific nodes or query results and the assigned colors to the Tree display.

Information displayed in tables can be exported from the application in XLS and Tab delimited formats.

#### Interactive distance matrix

**Figure 2.**
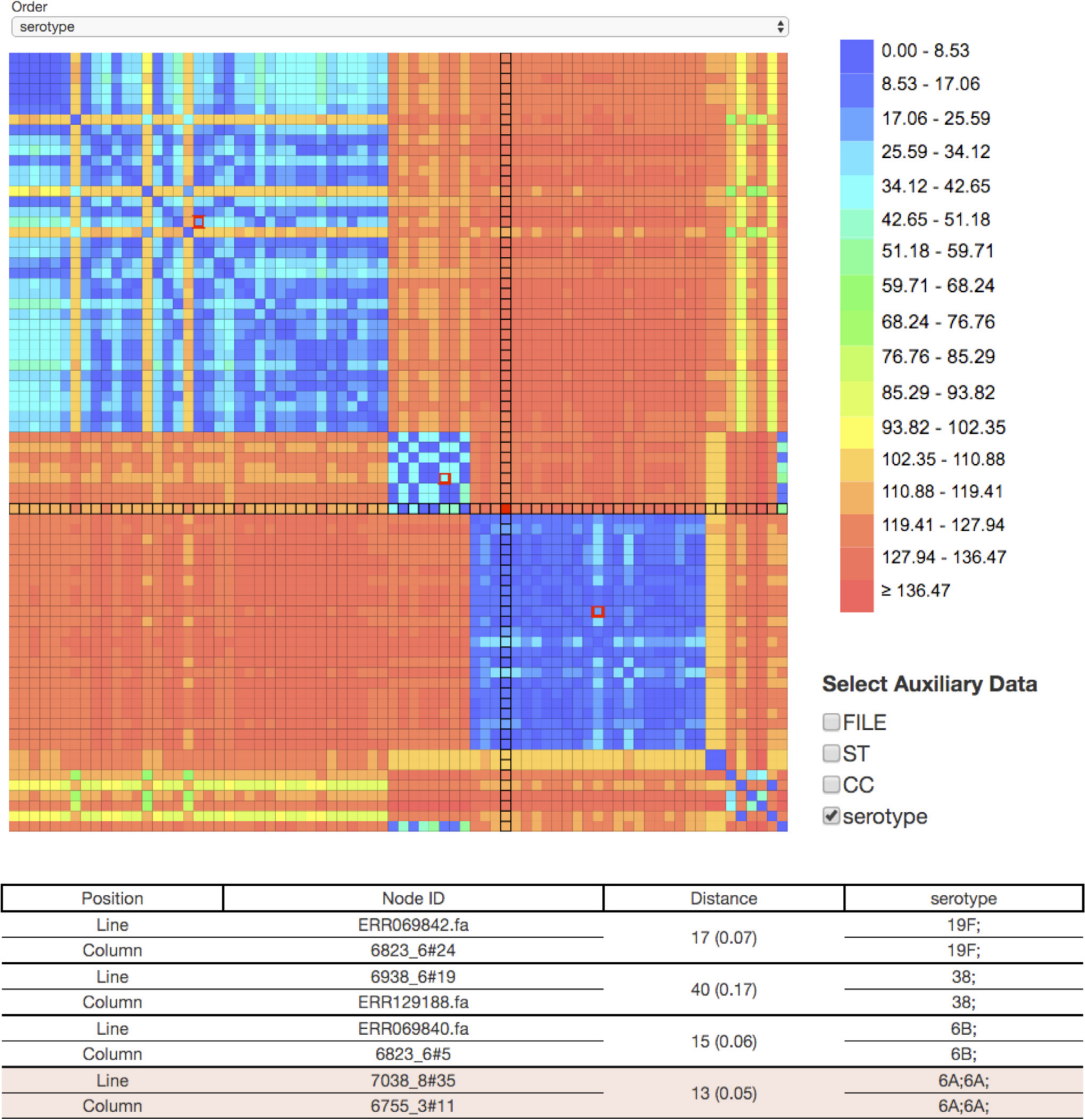
Interactive Distance Matrix constructed from a node selection of a cgMLST *Streptococcus pneumoniae* dataset. Matrix cells were ordered according to serotype and colors represent distances computed through pairwise comparisons of profiles. Selected nodes on the distance matrix (red border color) have their information displayed in the table according to the selected auxiliary data fields.

PHYLOViZ Online provides users with a dynamic Interactive Distance Matrix that can be constructed depending on the nodes that are selected in the Tree visualization tab (Figure 2).

After node selection and distance computation, the Distances tab is displayed. The tab is divided into two main areas: the interactive matrix and the information region. Colors in the distance matrix are attributed according to the number of differences between each pair of nodes, in case of Profile and FASTA input formats or according to the cladistics distance between nodes, for Newick files. On mouse over the matrix, the selected cell is highlighted and the chosen auxiliary information associated with it is displayed in the information region. Mouse clicking will store the data so that it can be exported as a Profile file and a tab-delimited file with the auxiliary data associated to the corresponding nodes.

The distance matrix can also be sorted according to the different fields present in auxiliary data, facilitating the visualization of relationships between strains sharing the same characteristics.

#### Sequence viewer

The application also offers a visual representation of multi-sequence alignment for FASTA input files. After node selection, clicking on the ‘View Sequences’ button in the Operations section opens the Sequences tab. In this visualization, the selected sequences are aligned, stacked and colored according to the sequence characteristics. Sequences can also be queried to find specific motifs, re-colored and ordered using the BioJS MSA Viewer package built-in options, and filtered according to a specific threshold.

### Data sharing

One goal of PHYLOViZ Online was to fill the existing software gap for sharing and reproducing phylogenetic inference using MST-like approaches. Users can have their datasets accessible to all by making them available in the Public datasets section. Another user will then be able to visualize and perform operations that do not change the dataset characteristics defined by the dataset owner. This option requires user registration in order to identify ownership and allow dataset modification and deletion by the owner. Additionally, users can allow others to access their datasets through the creation of dataset-specific URLs. By using this option, the user can limit access to the dataset to the group with whom the URL is shared. Access can be revoked at any time by the dataset owner. All datasets uploaded by unregistered users can also be shared through dataset-specific URLs but these will only be available for 24 h.

### RESTful API

PHYLOViZ Online also makes available an authenticated RESTful API, providing users with programmatic access to public data or to registered user data. Through the API, users can upload data and remotely run the goeBURST algorithm. The API was specifically designed to allow currently existing online databases storing sequence-based data to have a way to transfer data on the fly to PHYLOViZ Online in order to be able to offer data analysis capabilities directly on their stored data. API documentation and an example of usage are provided in the website (https://online.phyloviz.net/api).

### Example

An example dataset is provided on the web site so that users can directly interact with the application. Users can upload it or access it directly at https://online.phyloviz.net/main/dataset/public/cfa81215a2ce3fd3c8cb03c71d7ab342bf23fa8a9910ed7e/. A tutorial and an example video are also available at https://online.phyloviz.net. The dataset consists of 145 strains of *Streptococcus pneumoniae* whose partial genome sequence was publicly available (12,13). The allelic information of 237 loci in the genome was considered in an arbitrary cgMLST scheme. The auxiliary data considered was the capsular serotype information and the conventional MLST information (derived from the genomic data). The cgMLST profiles of all the strains differed at most in 107 loci (45% of all loci considered) and the two closest non-identical profiles at only two loci. Inspection of the Distance Matrix ordered by serotype reveals that strains sharing the same capsular serotype are more closely related (≤46 differences) than strains of different serotypes (≥110 differences). This occurs even if the strains were reported in different studies and recovered in different geographic locations. An exception to this is the strains of serotype 19F and 19A and strains of serotype 38 and those classified as reacting with pool I. Neither of these findings is surprising. Serotypes 19A and 19F are not only immunologically related but the loci encoding these capsular polysaccharides are also genetically related. On the other hand, one of the serotypes expected to be detected by the polyvalent pool I serum is 38, so these strains could in fact represent serotype 38. The close relationship between strains of the same serotype can be visualized on the tree by selecting the NLV graph with up to 23 differences (10% of the total number of genes considered, or a similarity of 0.9) that generates clusters of strains mostly expressing the same serotype. Inspection of the distance matrix reveals three strains expressing serotype 19F that are as different from other serotype 19F strains as these are from strains expressing distinct serotypes. Further investigation would be warranted to clarify if these strains result from capsular switching (acquisition of an heterologous capsular polysaccharide encoding locus).

## CONCLUSION AND FUTURE WORK

PHYLOViZ Online is freely available at https://online.phyloviz.net and provides an effective way for users to visualize, perform visual analytics and share annotated MSTs. While sharing ongoing analysis with more limited groups may facilitate restricted collaborations, publicly available datasets may function as permanent data repositories. As such, PHYLOViZ Online helps users to comply with the increasing requirement from journals to make available complete datasets and reproducible analyses that can be independently scrutinized. PHYLOViZ Online source code is freely available under GPLv3 license at https://github.com/bfrgoncalves/Online-PhyloViZ, so any user can set up their own PHYLOViZ web service. A set of Node.js modules is also available at https://www.npmjs.com/package/phyloviz_bundle for developers to incorporate PHYLOViZ tree visualization capabilities into their software. Future work will focus on novel network analysis methodologies and in the implementation of traditional tree visualization algorithms to complement the current force-directed tree display.
